# Strategic Integration of Surgical Techniques for Knee Preservation: A Case Report

**DOI:** 10.5704/MOJ.2507.016

**Published:** 2025-07

**Authors:** A Jaramillo-Quiceno, PA Sarmiento-Riveros, RD Arias-Perez, OA Mejia-Bustamante, RD Guzman-Benedek

**Affiliations:** 1 Department of Orthopaedic and Traumatology, Suramericana S.A. (EPS SURA), Medellin, Colombia; 2 Department of Orthopaedic and Traumatology, Clinica del Campestre, Medellin, Colombia; 3 Department of Orthopaedic and Traumatology, Pontifical Bolivarian University, Medellin, Colombia; 4 Department of Orthopaedic and Traumatology, Clinica El Rosario, Medellin, Colombia

**Keywords:** tibial meniscus injuries, knee joint, allogeneic transplantation, genu valgum, 3D model

## Abstract

Different surgical techniques are used to preserve knee articular cartilage deterioration; however, combining these techniques can be challenging. This case study describes a 35-year-old man with bilateral genu varum malalignment and left knee pain, diagnosed with a grade IV chondral lesion in the lateral femur, a complex lateral meniscus lesion, and a radial tear in the medial meniscus. The patient underwent a valgus-producing tibial osteotomy, lateral osteochondral allograft transplantation, and lateral meniscal allograft transplantation using a 3D model of the proximal tibia. Seventeen months post-operatively, he experienced torsional trauma, leading to a tear of the transplanted meniscus posterior root and an anterior cruciate ligament injury. Subsequent surgical exploration revealed evidence of chondroprotective changes, including femoral osteochondral allograft healing and biomechanical integration of the transplanted meniscus, as indicated by the lateral meniscus root tear development. This case highlights the potential of combining these surgical procedures to achieve biomechanical integration of the transplanted meniscus, contributing to cumulative chondroprotective effects.

## Introduction

The importance of the meniscus in normal knee function has been well documented. The meniscus decreases the tibiofemoral contact pressure and increases the load contact area, thus fulfilling a chondroprotective function^1^. Numerous studies have shown a greater susceptibility to early osteoarthritis (OA) after total meniscectomy^1^. To change the natural history of meniscal lesions, meniscal allograft transplantation (MAT) was developed and has gained popularity over the past decade^2^. There are many surgical techniques described for the MAT. Although there is controversy currently about which technique is best, the current trend is the bony fixation of the anterior and posterior horns^2^. Recent studies have shown that load transmission is superior when the meniscus is attached to the bone^2^. There is evidence of the chondroprotective effect of the simultaneous use of MAT and osteochondral transplantation as an articular cartilage preservation strategy^3^. In addition, MRI T2 mapping has been extensively used to confirm the chondroprotective effects of MAT and osteochondral allograft transplantation and provides a quantitative method for demonstrating the efficacy of therapies on articular cartilage^4^.

High tibial osteotomy (HTO) is an established procedure for the treatment of patients with medial compartmental OA and varus knee malalignment, particularly in young and active individuals. The medial opening-wedge HTO has become more popular because it is a simpler surgical technique that provides better control over the tibial slope^5^. This trend has been further supported by the development of fixation devices that enhance its usability. Traditional HTO aims to shift the mechanical axis of the knee from the medial to the lateral compartment to reduce the load on the medial side^5^. This change, moreover, may result in increased loading in the lateral compartment. Consequently, active patients with a varus knee lateral OA and lateral meniscus insufficiency have few treatment options.

For generally active, symptomatic patients who have complex, irreparable meniscal injuries, MAT has become a feasible alternative to partial or complete meniscectomy. Although there isn't much data to support the results of combining lateral MAT and medial opening-wedge HTO. Combining valgus-producing osteotomy, MAT, and articular cartilage surgery in the medial compartment has been shown to produce satisfactory results^1^.

To our knowledge, no prior studies have been published that combine the use of medial opening-wedge HTO, lateral MAT, and osteochondral allograft transplantation in the lateral femoral condyle because pathological genu varum and lesions in the lateral compartment are uncommon^3^.

## Case Report

A 35-year-old man presented to the outpatient clinic with mechanical pain in the left knee after suffering knee trauma 4 years ago while playing soccer. The patient did not seek medical attention at that time and did not receive any treatment. Since that trauma, he reported constant pain on the lateral side of the knee, which intensified when walking. On physical examination, a bilateral genu varum was observed, with a medial thrust gait, tenderness on the lateral side of the left knee, and positive meniscus tests such as Steinmann and McMurray's test for lateral meniscus. There was no tenderness or positive tests on the medial side. Anterior drawer, Lachman, Varus, and Valgus stress tests were also negative.

**Fig. 1:**
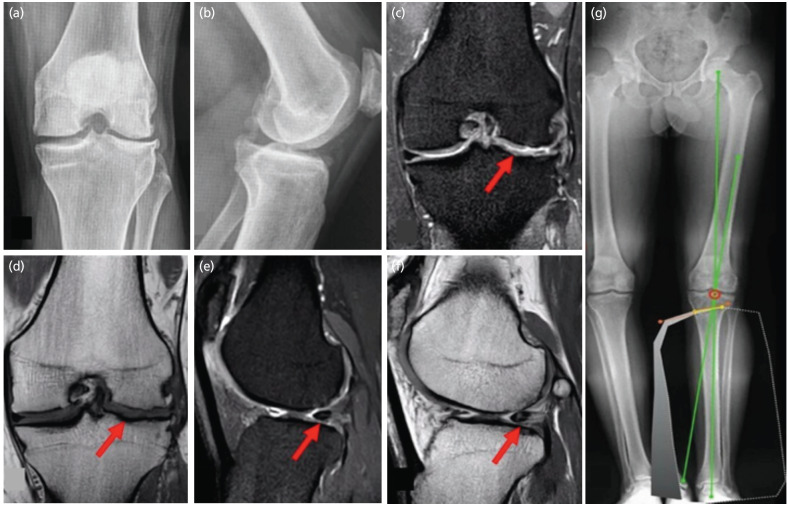
Pre-surgical diagnostic images. (a) Left knee radiograph AP view. (b) Lateral view with Kellgren-Lawrence classification OA grade III in the lateral compartment. (c) In pre-operative magnetic resonance imaging of the left knee, the red arrow indicates the chondral lesion in the lateral tibiofemoral compartment with femoral predominance in axial view with T1 sequence. (d) Fat-suppressed T2-weighted fast spin-echo sequence. (e) The red arrow indicates the medial meniscus posterior horn injury in sagittal view with T1 sequence. (f) Fat-suppressed T2- weighted fast spin-echo sequence. (g) Bilateral genu varum and surgical planning of tibial osteotomy. Left knee: Mechanical axis deviation (MAD) of 19mm, the mechanical lateral distal femoral angle (mLDFA) of 93°, medial proximal tibial angle (MPTA) of 87°, joint line convergence angle of 0°, correction angle of 7° (calculated to 50% of the tibial plateau), open wedge calculated of 6mm.

**Fig. 2:**
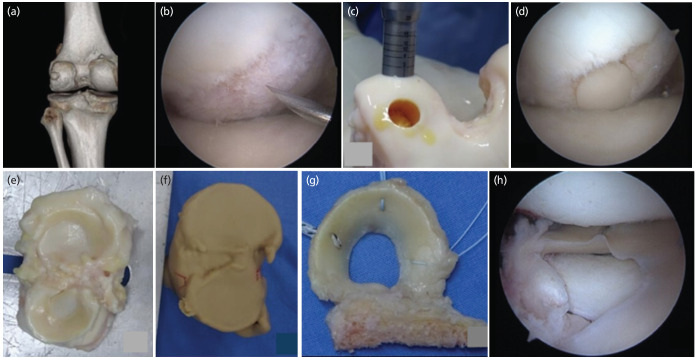
Intra-surgical findings and 3D model for surgical planning. Arthroscopic views and harvesting of osteochondral allografts. (a) Computed tomography of the left knee with 3D reconstruction. (b) Chondral lesion of the lateral femoral condyle in weight-bearing area. (c) Harvesting of osteochondral allografts. (d) Chondral lesion in the lateral femoral condyle covered by 80%. (e) Tibial plateau allograft. (f) Life-size 3D model of the proximal tibia. (g) Allograft preparation for meniscal transplantation using the dovetail technique. (h) Arthroscopic view of the transplanted lateral meniscus.

Subsequently, imaging tests were performed, including knee radiographs (Fig. 1a, b), where a Kellgren-Lawrence OA grade III classification was observed in the lateral compartment. Panoramic radiographs showed bilateral genu varum and a mechanical axis deviation (MAD) of 19mm in the left knee, in addition to a magnetic resonance image (MRI) (Fig. 1c-f), with a grade IV chondral lesion of the lateral femur according to the Outerbridge classification, as well as a non-suturable lateral meniscus injury and a radial tear of the medial meniscus. Besides, computed tomography (CT) to measure the tibial plateau and 3D life-size model printing for a better selection of the size of the meniscus transplant (Fig. 2a). The International Knee Documentation Committee (IKDC) score and the Knee injury and Osteoarthritis Outcome Score (KOOS) of the patient were 59 and 49, respectively.

It was decided to carry out at the same surgical time a lateral MAT, suture of the medial meniscus, lateral osteochondral allograft transplantation in the femur, and medial opening-wedge HTO seeking a neutral alignment (Fig. 1g). Tibial osteotomy was performed using a wedge of tricalcium phosphate [chronOS® Bone Graft Substitute] and TomoFix® medial high tibia plate fixed 7 locking screws and one cortical one. Subsequently, arthroscopy was performed, finding changes in the medial compartment with a grade II chondral lesion in the femoral condyle according to the Outerbridge classification, which underwent chondroplasty. In addition, a radial tear of the medial meniscus was managed with the all-inside meniscal suture technique using two stitches.

In the lateral femoral condyle, there was a grade IV chondral lesion of 22x13mm, in a weight-bearing zone (Fig. 2b). Fresh femoral condyle cartilage allografts were harvested and inserted with the 10mm COR™ cartilage transplant system in the femur (Fig. 2c), leaving the lesion 80% covered. (Fig. 2d). Subsequently, the fresh graft was prepared for the lateral meniscal transplant (Fig. 2e-g). The conventional dovetail technique was performed. Fig. 2h shows the result of the transplanted meniscus. The 3D model acts as a physical guide to assist the surgery.

**Fig. 3:**
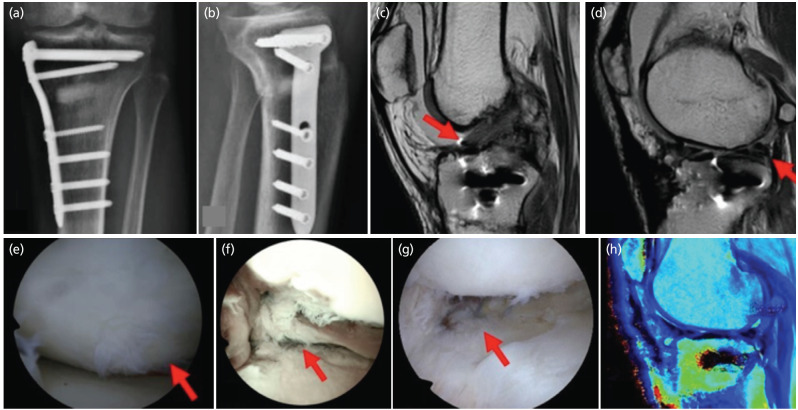
Clinical follow-up images. (a) Radiographs of the left knee four months post-operatively. Osteotomy hardware in an adequate position and evidence of bone healing. AP view. (b) Lateral view. (c) MRI after trauma with fat-suppressed T2-weighted fast spin-echo sequence. The arrow indicates the ACL injury. (d) The arrow indicates the injury to the posterior root of the transplanted meniscus. (e) Osteochondral graft healing in the lateral femoral condyle during the second surgical procedure. (f) Arthroscopic view, acute posterior root tear of the transplanted lateral meniscus during the second surgical procedure. (g) Sutured posterior root lesion of the lateral meniscus. (h) Post-operative sagittal view with MRI T2-mapping sequence, without an overload of the lateral compartment.

In the post-operative period, analgesia and thromboprophylaxis were ordered, allowing movement and maximum flexion of 90° in the first month, and walking no-weight bearing for the first 6 weeks using crutches. At 4 months post-operative, he presented an adequate range of motion of 0-110° and good alignment of the left knee, gait without limp, and the radiographs showed a progression in the consolidation of the osteotomy (Fig. 3a, b). At 12 months post-operative, the patient had a range of motion of 0-120° and improved IKDC and KOOS scores of 90 and 85, respectively.

A total of 17 months after the procedure, the patient suffered torsional trauma to the left knee, with joint pain and effusion. The MRI reported Anterior Cruciate Ligament (ACL) injury, osteochondral graft in the lateral femoral condyle with evidence of healing, and post-surgical changes of the transplanted lateral meniscus with posterior root tear (Fig. 3c, d). Therefore, it was decided to perform a new surgery to evaluate the meniscal injury and perform ACL reconstruction.

In the second surgical procedure, hardware removal of the proximal tibia was performed. During the arthroscopy, an osteochondral transplant of the lateral femoral condyle was observed with optimal healing (Fig. 3e), and the transplanted meniscus had adequate peripheral healing, but a posterior root tear occurred (an acute injury) (Fig. 3f). Posterior meniscal root repair was performed with transosseous tunnels in the tibia and two traction stitches using FiberLink® sutures (Fig. 3g), there was also another longitudinal meniscal lesion located near the popliteal hiatus, which was successfully sutured using an all-inside meniscal suture technique. Subsequently, ACL reconstruction was performed using an autologous hamstring graft.

In the post-operative period, adequate analgesia and thromboprophylaxis were ordered, and mobilisation was allowed as soon as post-operative pain allowed it, without flexion limitation, and walked no-weight bearing for the first 6 weeks using crutches. In the control after one month, he presented a range of motion of 0-90°. At 4 months postoperative, the patient presented improvement in range of motion of 0-110°, controlled pain, and gait without limp. At 6 months post-operative, an MRI was done, and T2 mapping revealed that there was no overload on the lateral compartment (Fig. 3h). At 12 months post-operative, the patient had a range of motion of 0-120°, and IKDC and KOOS scores of 87 and 82, respectively.

## Discussion

This case involved a patient with a pathological genu varum, a complex non-suturable lateral meniscus injury, a radial lesion in the posterior horn of the medial meniscus, and lateral compartment OA. Simultaneous medial opening-wedge HTO, lateral osteochondral allograft transplantation, medial meniscus suture, and lateral MAT were carried out. A life-size 3D model was used as a physical guide to aid in surgery and to obtain accurate measurements for graft selection for meniscus transplantation. The patient presented a satisfactory clinical evolution. However, 17 months after the procedure, the patient suffered torsional trauma to the left knee, which resulted in a lesion of the posterior root of the lateral transplanted meniscus and a tear of the ACL. This suggests biomechanical integration of the meniscal transplant, as similar injuries are commonly reported in native meniscus associated with ACL ruptures5, and it was possible to demonstrate chondroprotective changes, such as healing of the osteochondral allograft in the femur and no overload on the lateral compartment in the MRI T2-mapping. The case demonstrates the benefits of combining operations when articular cartilage preservation is the surgical aim.

The concomitant treatment of articular cartilage disease, meniscus deficiency, and malalignment is complex^5^. Particularly if there is varus-aligned lateral compartment knee osteoarthritis and irreversible lateral meniscus injury. Because a valgus-producing opening-wedge proximal tibial osteotomy may result in increased loading in the lateral compartment, it is necessary to correct the lower limb malalignment to be able to perform the meniscal transplant^3^. Furthermore, a crucial factor in the development of lateral compartment osteoarthritis and lateral meniscus injury is valgus alignment^5^. Therefore, the case presented is complex, and the best management options available were used, and a neutral alignment of the limb was performed to avoid lateral overload which was confirmed using MRI T2 mapping.

When there are adequate surgical indications to combine osteochondral allograft transplantation with meniscal transplantation, the clinical outcomes appear to be satisfactory. Despite the prolonged and more complex surgery, there is evidence that the osteochondral grafts in these cases survived sufficiently^3^. In the case presented, it was possible to demonstrate adequate healing of the osteochondral graft and a symptomatic improvement in the IKDC and KOOS scores during the follow-up. In addition to a satisfactory integration of the transplanted meniscus, the presence of a traumatic posterior root injury implies good biomechanical integration^3^, since traumatic injuries are closely related to the concomitant ACL injury, which occurred in this case.

This case is of special interest because the lesions found are uncommon, since varus knee alignment is associated with excess load and deterioration of the cartilage in the medial compartment, and these chondral lesions are usually chronic^1^. However, in the case presented, a certain degree of deterioration was evidenced in the medial compartment associated with the malalignment of the extremity, but the decline of the lateral cartilage was more significant in the clinical and radiological evaluation, it is due to the complex injury of the lateral meniscus. For this reason, it was decided to correct the varus malalignment with a tibial osteotomy, seeking a neutral alignment and avoiding overloading the lateral compartment, to achieve adequate healing of the lesions in this compartment. In addition, one of the great difficulties in performing a meniscal transplant is the proper selection of the graft size to be transplanted. The allograft must be properly sized to ensure the long-term success of the transplant since smaller grafts might cause the meniscus to tear and large grafts could improperly distribute the load^2^. Therefore, using a 3D model of the proximal tibia, such as the one presented in this case, helps select the appropriate graft size and acts as a physical guide to assist the surgery.

In conclusion, the case presented highlights that combining valgus-producing tibial osteotomy, lateral meniscal allograft transplantation, and lateral osteochondral allograft transplantation can effectively promote biomechanical integration and chondroprotective effects in patients with complex knee injuries. The case demonstrated satisfactory clinical outcomes, including successful healing of the osteochondral graft and functional improvement scores. The biomechanical integration of the transplanted meniscus was confirmed by the development of a posterior root tear and ACL injury following torsional trauma, which is characteristic of native meniscus injuries associated with ACL tears. The case also helps to illustrate the advantages of using 3D models of the proximal tibia to assist meniscal transplant cases and highlights the benefits and challenges of combining different surgical techniques for complex knee conditions.
